# Brain Activation During Processing of Depression Emotion in College Students With Premenstrual Syndrome in China: Preliminary Findings

**DOI:** 10.3389/fpsyt.2022.856443

**Published:** 2022-06-27

**Authors:** Mingzhou Gao, Li An, Yanhong Yu, Jieqiong Wang, Yanjiao Hou, Qiuqi Xu, Lvning Ren, Dongmei Gao

**Affiliations:** ^1^Team of Research and Innovation Focusing on Emotional Diseases and Syndromes, Innovation Research Institute of Traditional Chinese Medicine, Shandong University of Traditional Chinese Medicine, Jinan, China; ^2^Department of Traditional Chinese Medicine, Jinan Central Hospital, Jinan, China; ^3^Teaching and Research Office of Basic Theory of Traditional Chinese Medicine, College of Traditional Chinese Medicine, Shandong University of Traditional Chinese Medicine, Jinan, China; ^4^Scientific Research Achievements Transformation Department, Office of Academic Research, Shandong University of Traditional Chinese Medicine, Jinan, China; ^5^Medical Teaching Center, Open University of China Press Jinan Branch, Jinan, China

**Keywords:** PMS, task state, BOLD-fMRI, depression emotion, college students

## Abstract

**Background:**

This study aimed to investigate the neural substrates of processing depression emotion in premenstrual syndrome (PMS) and healthy subjects of college students using blood oxygenation level-dependent functional magnetic resonance imaging (BOLD-fMRI).

**Methods:**

During BOLD-fMRI scanning, 13 PMS patients and 15 healthy controls (HC) performed a picture visual stimulation task during luteal and follicular phases, in which participants and HC were asked to see pictures containing depression and non-depression emotions. Simultaneously, self-rating depression scales (SDS) were employed to evaluate the emotional status of participants.

**Results:**

Compared to HC, right inferior occipital gyrus, right middle occipital gyrus, right lingual gyrus, right fusiform gyrus, right inferior temporal gyrus, cerebelum_crus1_R, cerebelum_6_R, culmen, the cerebellum anterior lobe, tuber, and cerebellar tonsil of PMS patients showed enhanced activation. In contrast, sub-lobar, sub-gyral, extra-nuclear, right orbit part of superior frontal gyrus, right middle temporal gyrus, right orbit part of inferior frontal gyrus, limbic lobe, right insula, bilateral anterior and adjacent cingulate gyrus, bilateral caudate, caudate head, bilateral putamen, and left globus pallidus exhibited decreased activation.

**Conclusion:**

The findings indicate that abnormal functional regulation of brain regions such as occipital lobe and cerebellum leads to abnormal changes in emotional regulation, cognitive ability, and attention distribution in PMS patients, implying significant central pathogenesis.

## Background

Premenstrual syndrome (PMS) is a disorder that substantially impairs normal life activities and interpersonal relationships and is associated with a woman's menstruation cycle (1, 2). Premenstrual dysphoric disorder (PMDD) is a severe form of PMS (3). Established research indicated that PMS prevalence was 35.3% among Sharjah university students (4), 62.7% among Puducherry college students (5), 64.9% among female medical students in Saudi Arabia (6), and even higher in some regions. PMS causes various symptoms in women, commonly including affective symptoms, behavioral symptoms, and difficulty concentrating, impairing their quality of normal life (7, 8). Among them, women with PMS had difficulty in regulating their emotions (9), such as prominent depression (10), making them at higher risk of suicidality (11).

Although PMS pathogenesis remains unclear, the application of brain imaging technology has facilitated its intrinsic neural mechanism of neuropsychiatric disorders (12). Among them, blood oxygenation level-dependent (BOLD) functional magnetic resonance imaging (fMRI) has had a tremendous influence on human neuroscience over the last two decades (13). Qing Liu demonstrated decreased connectivity in the middle frontal gyrus (MFG) and parahippocampal gyrus (PHG) in PMS patients, as well as increased connectivity in the left medial/superior temporal gyri (MTG/STG) and precentral gyrus within the default mode network (DMN) using fMRI (14). Hai Liao revealed elevated regional homogeneity (ReHo) mainly in the bilateral precuneus, left inferior temporal cortex (ITC), right inferior frontal cortex (IFC), and left middle frontal cortex (MFC), as well as decreased ReHo in the right anterior cingulate cortex (ACC) of PMS patients during the luteal phase (15). Besides, structural MRI revealed increased gray matter (GM) volumes in precuneus/posterior cingulate cortex (precuneus/PCC) and thalamus, as well as decreased GM volumes in the insula of PMS patients (16). Concurrently, Demao Deng's research indicated that PMS patients have greater bilateral amygdalae volumes, increased FC between amygdala and certain regions of frontal cortex, the right temporal pole, and the insula, as well as decreased FC between bilateral amygdalae and right orbitofrontal cortex and right hippocampus (17). In addition, Peng Liu discovered decreased prefrontal-thalamic connectivity and increased posterior parietal-thalamic connectivity in PMS patients using resting-state fMRI (18).

Until now, few studies have been reported on the processing mechanism of depression emotion in PMS (3). Accordingly, this study aims to investigate the neural substrates of depression emotion processing in PMS using BOLD-fMRI.

## Materials and Methods

### Ethics Statement

The Medicine Ethics Committee of the First Affiliated Hospital of Shandong University of Traditional Chinese Medicine, Shandong, China, approved this study. All research procedures were conducted following the Declaration of Helsinki. All participants were apprised of the entire experimental procedure and signed an informed consent form.

### Participants

Thirteen right-handed PMS females were recruited to participate in this study and matched with a control group of 15 comparison subjects. In each group, subjects were matched according to their age and educational level. In addition, all subjects completed the Self-rating depression scale (SDS), which aims to determine depression severity. All subjects provided written informed consent.

### Inclusion and Exclusion Criteria

#### Inclusion Criteria for PMS

It firstly meets the international diagnostic standards for PMS of the American Society of Obstetrics and gynecology (ACOG). The subjects were college students, 20–25 years old, right-handed, and voluntarily participated in the study. Inclusion criteria also included good mental state, sleep quality, and appetite. Those who have clear consciousness, and independent judgment ability, can understand the purpose of this study and cooperate voluntarily. There are no major diseases such as heart, liver, and kidney, no brain tumor or other brain diseases, and no history of taking psychotropic drugs. Both eyes have a normal naked vision or corrected vision.

#### Inclusion Criteria for HC

The participants were healthy and had no history of nervous system diseases such as headache, dizziness, and seizures. And they were college students, 20–25 years old, right-handed, and voluntarily participated in the study. At present, the participants are in a good mental state, have good sleep quality and appetite, clear consciousness, independent judgment ability, able to understand the purpose of this study, and voluntarily cooperate with the experiment. The naked or corrected visual acuity of both eyes is normal.

#### Exclusion Criteria

Participants will be excluded if they are mentally ill, have serious physical diseases, have a history of drug abuse (including drugs used to treat PMS within 3 months), have blood system diseases, or have aphasia, disturbance of consciousness, dementia, and other conditions that cannot cooperate with the examination, have a chronic history of five visceral diseases such as heart and liver and have clinical symptoms, or undergo unilateral oophorectomy or abortion within 6 months, take contraceptives, or the head translation monitored during motion correction exceeds 3 mm; or rotate more than 1 degree in any direction in the three-dimensional direction. Those who put metal objects in their bodies (including pacemakers, metal dental materials, wearing braces, etc.) were excluded.

### Stimuli Paradigm

The picture visual stimulation task consisted of depression and neutral emotional pictures filtered by the international emotional picture library. Stimuli were presented using the experimental visual stimulus program (Electronic Technology in Medicine Co., Ltd., Shenzhen, China). Each stimulus onset (masked face or crosshair) was triggered directly by a pulse from the scanner. The images were projected onto a computer screen behind the subject's head within the imaging chamber. The screen was viewed by a mirror positioned ~8 cm above the subject's face.

**Figure 1:**
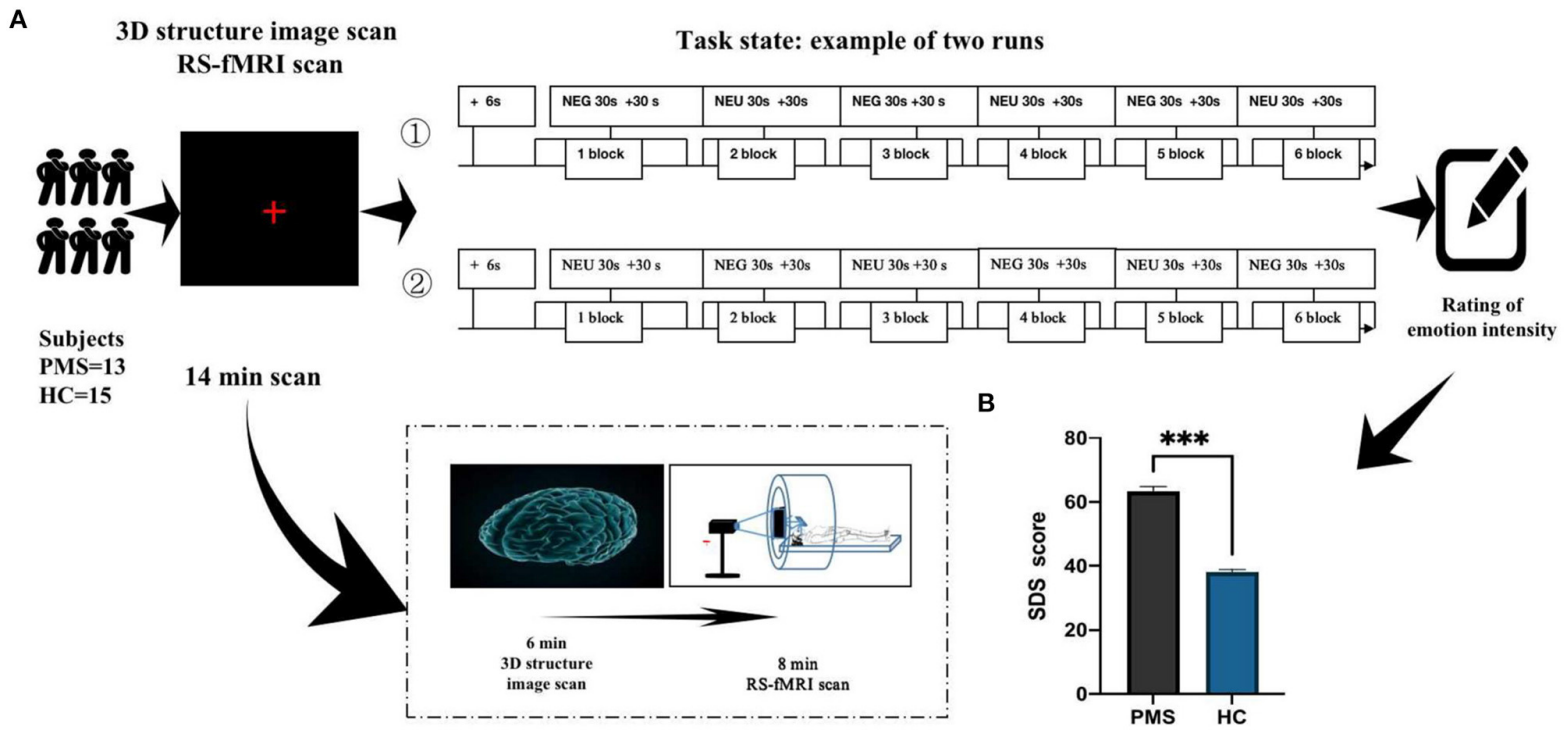
Timeline of experiments (fMRI). **(A)** Demonstrates the overall operating flow of emotional stimuli experiments during fMRI. **(B)** Illustrates depression degree in PMS patients after depression picture stimulation during the luteal phase of the menstrual cycle. ^***^P < 0.00.

During fMRI, all subjects were shown depression and neutral emotional pictures in a block design [See Figure 1A Examples of depression pictures from the International Affective Picture System (IAPS) chosen referring to previous study (19)]. The task state contains two runs and six blocks. Each block had neutral emotional pictures interspersed with emotional pictures in a pseudo-random order. This ensured that emotional pictures occurred unpredictably. Depression emotional picture stimulus consisted of a 30-s presentation (each picture was presented for 5 s, six pictures in a block), followed by a 30-s presentation of neutral pictures (each picture was presented for 5 s, six pictures in a block). The subject saw 30 negative emotional pictures within the emotional stimuli blocks in a predetermined random order. The subject also saw 30 neutral emotional pictures within the neutral picture blocks in a predetermined random order. Picture stimuli were presented at a rate of one per echo-planar image (EPI) sequence. Following each face block, a control period of 30 cross-hair stimuli fixation points (+) was presented at the same rate as the emotional pictures (see Figure 1).

The presentation order of emotional pictures was identical for all subjects across runs. The first run consisted of + Negative emotion pictures (NEG) + Neutral emotion pictures (NEU). The second run was + NEU + NEG. Each run lasted 6 min. Each subject viewed two runs. Following the scan, subjective reports of pictures evoking emotional effects were evaluated using SDS. Higher scores indicated that subjects experienced higher emotional strength. Subjects must carefully choose while assessing emotional intensity, which may induce different emotions.

### Image Acquisition and Data Analysis

Functional magnetic resonance imaging images were obtained on a 3.0-T MR scanner equipped with a prototype fast gradient system for echo-planar imaging (EPI) at the Institute of Medical Imaging of Shandong. Functional images were obtained using an echo-planar imaging sequence with the following parameters: TE = 35 ms; TR = 2,000 ms; slice thickness = 4 mm; gap = 1 mm; flip angle = 90°; FOV = 24 cm; and in-plane resolution = 64 × 64.

Functional MRI data were preprocessed using Statistical Parametric Mapping (SPM8). We discarded data of subjects whose head motions of more than 3.0 mm maximum displacement in *X, Y*, or *Z* directions or 2.5 degrees in any angular direction. The first three volumes of functional images were discarded due to signal equilibrium and participants' adaptation to the scanning noise. For each participant, functional images were realigned using least-squares minimization without higher-order corrections for spin history and were normalized to Montreal Neurological Institute (MNI) template from structural images. Images were re-sampled to 3 × 3 × 3 mm^3^ and smoothed with a 6-mm full-width at half maximum.

### Statistical Analysis

Individual data were analyzed by creating a generalized linear model (GLM) in SPM. First level analysis was performed using a General Linear Model [GLM, (53)] applied to the time series, and convolved with the canonical hemodynamic response function. A high pass filter of 128 seconds was applied in order to remove slow signal drifts and improve signal to noise ratio. For each emotional condition, two conditions were defined: depression emotional pictures and neutral emotional picture. In GLM analysis, when setting the model matrix, NEG vs. NEU, the block in NEG is set to 1, and the block in NEU is set to −1. When looking at the main effect of NEG alone, the trail in NEG is set to 1, and the rest are set to 0. Whole-brain voxel-based activation analysis was used to calculate the activation strength in each voxel in each subject and convert it into con-maps (con-maps is a contrast file, which represents the comparison operation of beta values under different conditions).Group-level statistical analyses were performed using a random-effects model in SPM8. Two-sample *t*-test was conducted on the individual con-maps of the two groups with small volume correction for the one sample results masks. The volume threshold for each cluster was >389 consecutive voxels; the single voxel threshold for brain regions was *P* < 0.05 (corrected). Multiple comparison correction for the results was performed using simulation (see program AlphaSim by B.D. Ward, http://afni.nimh.nih.gov/pub/dist/doc/manual/AlphaSim.pdf), with a statistically significant difference. A double-sample *t*-test was used to analyze the case and control groups.

## Results

### Demographics

This study included 13 women with PMS and 15 matched HC. The sample size was determined prospectively and was bigger than existing published studies evaluating brain activity in PMDD women (20–22). The groups did not differ significantly in age (years), menophania (years), length of menstrual cycles (days), menstruation (days), all *p*s > 0.1 (see Table 1).

**Table 1:** Demographics of PMS and HC groups.

**Variables**	**PMS (*n* = 13)**	**HC (*n* = 15)**	***P*** **-value**
Age (years)	24.421 ± 0.838	24.762 ± 1.338	0.346
Menophania (years)	13.737 ± 1.240	13.476 ± 0.981	0.463
Length of menstrual cycles (days)	6.211 ± 1.228	5.714 ± 1.189	0.202
Menstruation (days)	31.053 ± 2.483	30.286 ± 2.077	0.294

*All values are mean ± standard deviation (SD)*.

### Degree of Depression

After the subjects completed the experiment, they were asked to identify depression severity using SDS. Depression degree in PMS patients was significantly higher than that in the HC group (*P* < 0.001; Figure 1B).

### Group Differences in BOLD-fMRI

**Figure 2:**
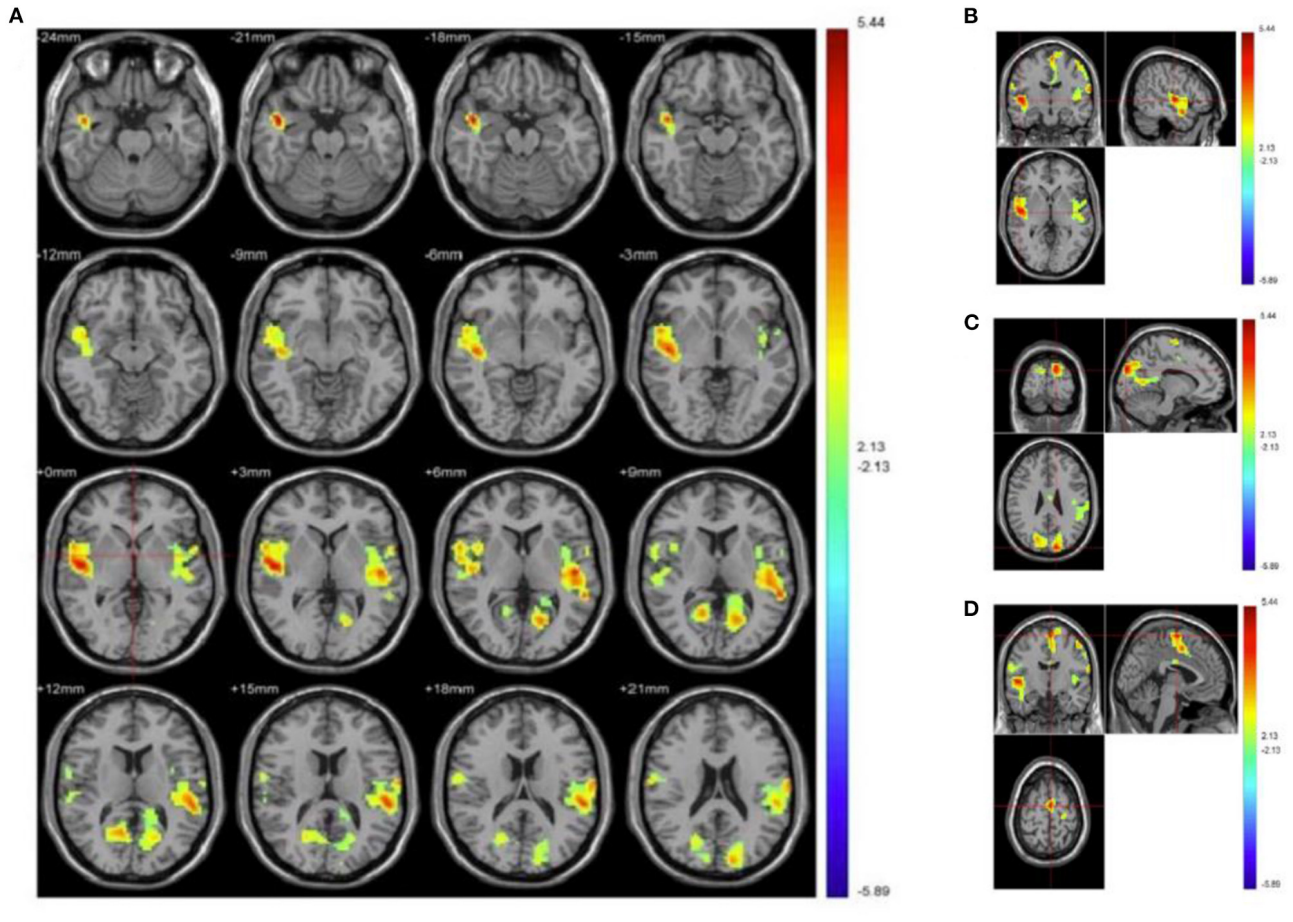
Increased and decreased activation of PMS patients' brain regions than healthy controls when processing depression vs. neutral pictures. **(A)** Depicts overall activation of the brain area by negative emotional picture task in PMS patients. **(B)** Manifests bilateral temporal lobe. **(C)** Illustrates bilateral precuneus, posterior cingulate, and cuneiform. **(D)** Demonstrates the brain areas activated in motor areas. Red indicates enhanced activation of brain regions in PMS patients, while blue indicates that activation is decreased.

Compared with HC group, PMS patients exhibit increased activation in the following brain regions: right inferior occipital gyrus, right middle occipital gyrus, right lingual gyrus, right fusiform gyrus, right inferior temporal gyrus, cerebelum_crus1_R, cerebelum_6_R, culmen, cerebellum anterior lobe, tuber, and cerebellar tonsil. Simultaneously, PMS patients have decreased activation of the following brain areas: sub-lobar, sub-gyral, extra-nuclear, right orbit part of superior frontal gyrus, right middle temporal gyrus, right orbit part of inferior frontal gyrus, limbic lobe, right insula, bilateral anterior and adjacent cingulate gyrus, bilateral caudate, caudate head, bilateral putamen and left globus pallidus, (Figure 2 and Table 1).

## Discussion

According to established research, women with PMS appear to experience emotional dysregulation throughout the menstrual cycle (23). Additionally, students experienced more emotional regulation deficits (24). Among premenstrual symptoms, depression was the most prominent feature of PMS diagnosis and should be properly evaluated and treated (10). Our findings indicated that the depression degree in PMS patients was significantly higher than in the HC group (Figure 1B), which is consistent with our findings in PMDD (19). Women with PMS/PMDD show significant depression, which is a response to abnormal changes in the brain. When PMS patients are exposed to emotional stimuli, the function of the spindle gyrus in occipital and temporal lobes, as well as activation of the right infratemporal gyrus and cerebellum, are enhanced, while sub lobar, sub gyral, extra nucleus, frontal, marginal, and basal nuclei are weakened (Table 2 and Figure 2). It demonstrates that the above-mentioned brain area function regulation is abnormal before menstruation, followed by emotional, cognitive, and attention distribution changes, all of which are associated with PMS pathogenesis.

**Table 2:** Difference area of two sample *t*-test under the condition of subtracting neutral picture from negative emotion picture between PMS group and HC group in BOLD-fMRI.

**Brain region**		**Peak MNI coordinate**			**BA**	***T*** **-Value**	**Sub-brain region**	
**Clusters peak voxel**	**Cluster voxels**	**X**	**Y**	**Z**			**Sub-cluster**	**Sub-cluster voxels**
Putamen_L	1,418	−12	6	−9	BA 25	−4.3294	Orbit part of superior frontal gyrus	105
							Middle temporal gyrus	67
							Orbit part of inferior frontal gyrus	57
							Rectal gyrus	73
							Sub-lobar	289
							Sub-Gyral	124
							Extra-nuclear	113
							Right insula	22
							Amygdala hippocampus	142
							Anterior and adjacent cingulate gyrus	47
							Caudate	107
							Caudate head	78
							Putamen	126
							Caudate	45
							Globus pallidus	23
Cerebelum_Crus1_R	642	39	−75	−18	BA 19	3.7694	Cerebelum_Crus1_R	123
							Cerebelum_6_R	60
							Culmen	54
							Cerebellum anterior lobe	54
							Tuber	35
							Cerebellar tonsil	29
							Right inferior occipital gyrus	111
							Lingual gyrus	12
							Right fusiform gyrus	104
							Fusiform gyrus	32
							Inferior temporal gyrus	28

*BA, Brodmann's areas*.

Our findings corroborate other research conducted both domestically and internationally. The frontal lobe is involved in spiritual activities associated with an individual's emotions (25). The prefrontal cortex (PFC) plays a critical role in emotion generation and regulation (26). Furthermore, the prefrontal cortex edge, especially the orbitofrontal cortex, influenced decision-making and emotional regulation (27). When untreated depression patients viewed negative emotional stimuli, the right orbitofrontal cortex (28) (middle frontal gyrus) oxygen-dependent reaction weakened, which may be linked to depression emotion.

Besides, insular is involved in emotional processing and influences individual decisions (29). In the task state, insula and insular cortex activity of PMDD patients significantly increased during the luteal phase (29). Our findings revealed that right insula activation decreased in task state, which is a new discovery in PMS research that is not identical to PMDD (19). Additionally, there were changes in the hippocampus cortex of PMDD patients (30). Amygdala, hippocampus, and anterior cingulate belong to the limbic lobe, which is intimately connected to emotional, functional activities (31). Cerebellum was linked to cognitive function (32). PMDD subjects had greater cerebral gray-matter volume than controls in the posterior cerebellum (33). The cerebellar activity of PMDD patients increased from follicular phase to late luteal phase (34), especially cerebellar vermis, which was correlated with emotional deterioration, as confirmed by our study. Additionally, our findings indicated that culmen, cerebellum anterior lobe, tuber, and cerebellar tonsil were intimately associated with PMS.

### Limitations

For now, our findings in college students with PMS in China have suggested their basic neural mechanism, and we need to aim at the deeper mechanism of PMS/PMDD and explore correlation between BOLD fMRI and SDS in the future study. Besides, the physiological components induced by heart rate and respiration were not considered in our study. We will pay more attention to the analysis of influencing factors such as heart rate.

## Conclusions

In summary, PMS's abnormal brain regions were localized using BOLD-fMRI in college students, indicating pathological brain changes. However, these new findings must be confirmed and replicated in the future using larger sample size and animal models.

## Data Availability Statement

The raw data supporting the conclusions of this article will be made available by the authors, without undue reservation.

## Ethics Statement

The studies involving human participants were reviewed and approved by Medicine Ethics Committee of the First Affiliated Hospital of Shandong University of Traditional Chinese Medicine. The patients/participants provided their written informed consent to participate in this study. Written informed consent was obtained from the individual(s) for the publication of any potentially identifiable images or data included in this article.

## Author Contributions

DG and MG designed the study, wrote the draft, and revised it. MG and YH performed the experiment. MG, QX, and LR collected data. MG performed the statistical analyses and finally edited manuscript. YY, LA, and JW provided key assistance. All authors contributed to and have approved the final manuscript.

## Funding

This study was sponsored by Key project of Natural Science Foundation of Shandong Province (ZR2020ZD17), National Natural Science Foundation of China (81001484; 81473558), Natural Science Foundation of Shandong Province (ZR202102270167), Shandong medical and health science and technology development plan project (202105010467) and 20 articles for colleges and universities funded project in Jinan (2020GXRC002).

## Conflict of Interest

The authors declare that the research was conducted in the absence of any commercial or financial relationships that could be construed as a potential conflict of interest.

## Publisher's Note

All claims expressed in this article are solely those of the authors and do not necessarily represent those of their affiliated organizations, or those of the publisher, the editors and the reviewers. Any product that may be evaluated in this article, or claim that may be made by its manufacturer, is not guaranteed or endorsed by the publisher.

## Abbreviations

PMS: premenstrual syndrome
HC: healthy control
BOLD-fMRI: blood oxygenation level-dependent functional magnetic resonance imaging
ACOG: American Society of Obstetrics and Gynecology
SDS: self-rating depression scales
MFG: middle frontal gyrus
PHG: parahippocampal gyrus
MTG/STG: left medial/superior temporal gyri
DMN: default mode network
ReHo: regional homogeneity
ITC: inferior temporal cortex
IFC: inferior frontal cortex
MFC: middle frontal cortex
ACC: anterior cingulate cortex
GM: gray matter
PCC: posterior cingulate cortex
IAPS: International Affective Picture System
EPI: echo-planar image
MNI: Montreal Neurological Institute
NEG: negative emotion picture
NEU: neutral emotion pictures.
